# Self-contained system for mitigation of contaminated aerosol sources of SARS-CoV-2

**DOI:** 10.21203/rs.3.rs-237873/v1

**Published:** 2021-02-24

**Authors:** Bhavesh Patel, Erica Forzani, Amelia Lowell, Kelly McKay, Karam Abi Karam, Adithya Shyamala Pandian, Gabriel Pyznar, Xiaojun Xian, Michael Serhan

**Affiliations:** 1 Department of Critical Care Medicine, Mayo Clinic, Phoenix, Arizona, USA; 2 School of Engineering for Matter, Transport, and Energy, Arizona State University, Arizona, USA; 3 Center for Bioelectronics and Biosensors, Biodesign Institute, Arizona State University, Arizona, USA; 4 School of Electrical, Energy and Computer Engineering, Arizona State University, Arizona, USA; 5 Department of Respiratory Care, Mayo Clinic, Phoenix, Arizona, USA; 6 Center for Military Medicine, Mayo Clinic, Phoenix, Arizona, USA

## Abstract

Contaminated aerosols and micro droplets are easily generated by infected hosts through sneezing, coughing, speaking and breathing^1–3^ and harm humans’ health and the global economy. While most of the efforts are usually targeted towards protecting individuals from getting infected,^4^ eliminating transmissions from infection sources is also important to prevent disease transmission. Supportive therapies for Severe Acute Respiratory Syndrome Coronavirus 2 (SARS CoV-2) pneumonia such as oxygen supplementation, nebulizers and non-invasive mechanical ventilation all carry an increased risk for viral transmission via aerosol to healthcare workers.^5–9^ In this work, we study the efficacy of five methods for self-containing aerosols emitted from infected subjects undergoing nebulization therapies with a diverse spectrum on oxygen delivery therapies. The work includes five study cases: *Case I:* Use of a Full-Face Mask with biofilter in bilevel positive airway pressure device (BPAP) therapy, *Case II:* Use of surgical mask in High Flow Nasal Cannula (HFNC) therapy, *Case III:* Use of a modified silicone disposable mask in a HFNC therapy, *Case IV:* Use of a modified silicone disposable mask with a regular nebulizer and normal breathing, *Case V:* Use of a mitigation box with biofilter in a Non-Invasive Positive Pressure Ventilator (NIPPV). We demonstrate that while *cases I, III* and *IV* showed efficacies of 98–100%; *cases II* and *V*, which are the most commonly used, resulted with significantly lower efficacies of 10–24% to mitigate the dispersion of nebulization aerosols. Therefore, implementing *cases I, III and IV* in health care facilities may help battle the contaminations and infections via aerosol transmission during a pandemic.

The coronavirus (COVID-19) pandemic has already infected over 45 million across the world and is responsible for more than 1.1 million deaths as of November 1st 2020.^10^ The disease is caused by the Severe Acute Respiratory Syndrome Coronavirus 2 (SARS-CoV-2)^11^. The mortality rate is estimated to be nearly 5% for those between the ages of 45–64 years old and a striking 19.1% for those in the 75+ age group^12^. Clearly, SARS-CoV-2 is a massive risk given the widespread transmission of the virus.

To date, there is a plethora of evidence demonstrating that SARS-CoV-2 transmission occurs fundamentally from the spread of viral pathogens in the infected host’s respiratory system to other susceptible hosts in contact with droplets, aerosols and fomites.^3, 7, 13, 14^ An aerosol is a suspension of fine particles (which can include viral pathogens) in an airborne liquid mist and can be transported through ventilation systems (e.g. AC) since it is not strongly affected by gravity^3^. Since SARS-CoV-2’s effective size is ~100 nm^11, 15^, it can be encapsulated within aerosols from the respiratory system of an infected person, presenting a major health risk to the environment of any building where a SARS-CoV-2 infected person might reside or be present^1, 3^. Ninety-nine percent of aerosols produced by humans, regardless of age, sex, weight and height are less than 10 μm^2, 3^. This is concerning since the smaller the aerosol, the longer it takes to settle increasing the risk of inhaling the contaminated aerosols by other individuals^16^. For example, an 0.5 μm aerosol takes 41 hours to settle^17^. In case of SARS-CoV-2, the viruses can be viable on a surface for up to 3 days^18^. Researchers have found SARS-CoV-2^14^ with virulent activity^3^ in collected aerosol particles from 0.2 μm to 10 μm, which is a serious concern for the spread of the disease through air conditioning (AC) systems^7, 19^. More recently CDC has recognized the importance of the transmission potential of SARS-CoV-2 via aerosols^20^. *The groundbreaking evidences strongly indicates the need of aerosol mitigation to safeguard the public and public spaces of the populations.*

In the present work, we target the mitigation of aerosol dispersion during nebulization treatments to provide a means of safe treatment for a respiratory disease such as COVID-19. This treatment causes a high risk of spreading pathogens due to the nebulization therapy generating aerosols with sizes less than 10 μm^21^, with emphasis on aerosol portions that do not impact the alveolar area, but remain in the dead space of the respiratory system (including nose and mouth) in contact with infectious areas and are exhaled into the environment^9^. Fig. 1a illustrates the problem by showing aerosol particle counts / feet^3^ transient over time inside a room with a COVID-19 patient during a nebulization therapy. The measurement was carried out in a Room of 17’ × 13.6’ × 9’, equipped with a small bathroom of 4’ × 6.25’ × 9’ in the back and an air ventilation rate, which is purposely set at ~20 air changes per hour^−1^ to minimize transmission of the disease via aerosols^17, 19^. The measurement was performed with a total of 3 aerosol sensors located at 3 (DL-1), 6 (DL-2) and 9 feet (DL-3) away from the subject as shown in Fig. 1b. The sensors were able to detect aerosol particles equal to or larger than 0.19 μm (see **Supplementary Information, Fig. 1**). The COVID-19 patient was assisted through an oxygen delivery therapy via a high flow nasal canula (HFNC) at 60 L/min. A nebulization therapy and an exercise therapy were delivered to the patient under the supervision of a respiratory therapist during the measurement. The nebulization included a total 3 ml solution containing 2.7 ml of Albuterol and 0.3 mL of saline physiological solution delivered through a Piezoelectric Based Nebulizer^22^. The subject did not wear any mask to mitigate the aerosol dispersion. As it can be observed, significant particle counts / feet^3^ were detected above the baseline level of the room, typically at ~ 7,400 counts / feet^3^ (for 0.2 μm - 5μm size count). During nebulization, the peak level was 365 times greater than the baseline level, and the particles clear out from the room 20 min after the completion of the nebulization. In addition, the proactive respiratory therapy executed by a therapist and patient’s breathing exercises under various conditions did not produce detectable aerosol-particle counts. Further, the action of flushing the bathroom toilet did not produce a particle count on the closest particle counter located 6 feet from the bathroom since the bathroom was small and had its own ventilation system with air exchange rate of ~20 h^−1^. *This seminal data indicates the nebulization therapies in COVID-19 patients should be targeted as a main source of potential contaminated aerosol in the environment.*

Aerosol spreading patterns are dependent on air exchange rates and ventilation streams of the room, the mechanism of nebulizers, and environmental (e.g. temperature and humidity) conditions. In fact, Fig. 1a indicates that the spatial distribution of the aerosol in the room is counterintuitive. Although the aerosol concentration at 3 feet was the highest, the peak concentration at 9 feet was 1/3 larger than the corresponding peak at 6 feet location. In order to solve the problem of highly variable and unpredictable aerosol patterns, we mitigate the dispersion by *DIRECTLY attacking the problem at the point of contaminated dispersion source and studying the effect of different mitigation systems that could potentially self-contain nebulizer aerosol dispersions*. Fig. 2a–b shows a summary of the efficacy of different mitigation systems that are applied to different oxygen therapies and nebulizers. The mitigations systems include 1- a biofilter which is a bacterial/viral filter with a humidity exchanger, 2- a silicone disposable mask (Breezing™) modified with a biofilter and a built-in fan, 3- a surgical mask, 4- a mitigation box with a biofilter. The application of the mitigation methods to different oxygen therapies rendered five study cases, as follows:

*Case I:* Use of a Full-Face Mask with biofilter in bilevel positive airway pressure device (BPAP) therapy (Fig. 3a–I), *Case II:* Use of surgical mask in High Flow Nasal Cannula (HFNC) therapy (Fig. 3a–II), *Case III:* Use of a modified silicone disposable mask in a HFNC therapy (Fig. 3a–III), *Case IV:* Use of a modified silicone disposable mask with a regular nebulizer and normal breathing (Fig. 3a–IV), *Case V:* Use of a mitigation box with biofilter with BiPAP Non-Invasive Positive Pressure Ventilator (NIPPV) (Fig. 3a–V). The efficacy of the nebulization therapy mitigation is assessed using the peak and the area under the aerosol particle counts / feet^3^ transient curves for both the no mitigation and mitigation cases. As an example, Fig. 2c shows the aerosol particle counts / feet^3^ transient over time at 3 feet from a patient during a nebulization therapy with and without the mitigation system. The following equations are used in connection with transients as the ones shown in Fig. 2c for calculation of the peak and area efficacy of the systems:

(Eq. 1) PeakEfficacy(%)=[(Peakwithnomitigationsolution)−(Peakwithmitigationsolution)]/Peakwithnomitigationsolution

(Eq. 2) AreaEfficacy(%)=[(Areawithnomitigationsolution)−(Areawithmitigationsolution)]/Areawithnomitigationsolution

The peak and area efficacies are evaluated for curves taken at 3, 6 and 13 feet with experimental setup similar to the one shown in Fig. 1b and averaged to report the efficacy registered for the particular condition. **Supplementary information (SI), Fig. 4–8** show the corresponding particle counts / feet^3^ profiles obtain for *Case I* (**SI, Fig. 3**), *Case II* (**SI, Fig. 4**), *Case III* (**SI, Fig. 5**), *Case IV* (**SI, Fig. 6**), and *Case V* (**SI, Fig. 7**). As it can be observed in Fig. 2a–b, *Case II* and *Case V*, which includes a surgical mask and a mitigation box, have only a mitigation efficacy of 10–24% to capture of nebulizer aerosols. Unfortunately, these have been commonly used methods to mitigate nebulization’s aerosol dispersions. On the contrary, *Cases I*, *III* and *IV* render an efficacy of 98–100% to capture of nebulizer aerosols. Since HFNC and BPAP are the preferred oxygen therapies to delay need for invasive mechanical ventilation in COVID-19 patients, *Cases I* and *III* includes representative solutions to mitigated aerosols during nebulization of COVID-19 patients or any patient with an infectious respiratory disease. Further, in order to produce 100% efficacy in *Case I*, it is observed that the complete sealing of the Full-Face Mask is required, given that any leak generates aerosol release (**Supplementary information (SI), Fig. 4A–C**). *Cases III* and *IV* include the use of a modified silicone disposable mask which has: 1- an inlet port for the delivery of the oxygen delivery therapy, 2- an outlet port for eliminating the residual aerosols from the therapy together with the unused air from the therapy, which is equipped with the filtering membrane and the fan, and 3- a safety/emergency inlet port with a one-way valve for inhalation of ambient air to be used in case the oxygen therapy stops. The mask incorporates the synergic action of convective transport from a *fan* and the filtration process from a *biofilter with bacterial and viral filtering membrane* of 0.2 μm pore size and multiple layers of microfiber material (Fig. 3b). In addition, it features an ergonomically designed silicone edge that enables adaptation to the user’s face, which is essential for a good seal to prevents leakage of contaminated air. The fan allows the driving of the aerosols through the biofilter, assuring all aerosols of 0.2 μm and larger are captured before the main air stream of the therapy flow exists the system to the atmospheric environment. It is driven by a 5V portable and rechargeable battery that can sustain the fan’s driving under the system’s planned operative conditions for ~6 hours continuously. Considering a nebulization therapy (including nebulization + therapy) takes 20 minutes, the battery can sustain 12 therapies.

In summary, we demonstrate here existing masks and boxes mitigation methods are inefficient for capturing aerosols generated from nebulizers. On the contrary, we probed the high efficacy for nebulization aerosol capture of *a biofilter used in conjunction with a fully sealed Full-Face Mask (FFM*) in **BiPAP** therapy and *the modified silicone disposable mask* in **HFNC** therapy and **Normal Breathing**. Given the crisis the whole world is facing with SARS-CoV-2 spread, the highly efficient aerosol trapping systems can help to limit transmission of the airborne virus in the current COVID-19 epidemic or other current respiratory infectious disease pathogens or future pandemics.

## Methods

[Will appear in online pdf and full-text version online. Do not exceed 3000 words. Subdivide using headings, continue references from above]

### Aerosol sensing instruments

The particles and aerosols generated during the nebulization procedures are assessed using the following commercial optical particle counters: MET ONE HHPC2+ from Beckman Coulter, and Dylos DC1100 Pro and Dylos DC1700 from Dylos Corporation, CA. MET ONE HHPC2+ particle counter provides reading for two ranges of particle sizes: 0.5 μm and 5.0 μm, which typical correspond to measuring particles ≥0.5 μm and ≥5.0 μm, respectively. Dylos DC1100 Pro and Dylos DC1700 particle counters provide readings for particle sizes of 0.5 μm and 2.5 μm, which typical correspond to measuring particles ≥ 0.5 μm and ≥ 2.5 μm, respectively. The sensors from Dylos DC1100 Pro and Dylos DC170 have identical sensing chambers but differ in the way they are powered. While Dylos DC1100 Pro is powered with a power adapter, Dylos DC1700 can be battery operated.

To decide on the best particle counters for this study, two additional particle counters were evaluated. These include: Dylos DC1100 from Dylos Corporation, CA with a capacity to detect to two ranges of particles: 1.0 μm and 5.0 μm (representing detection to ≥ 1.0 μm and ≥ 5.0 μm, respectively), and Dusttrak DRX aerosol monitor from TSI Incorporated with a capacity to detect Aerosol concentration range 0.001 to 150 mg/m^3^.

### Verification test of the aerosol sensing instruments

**Figure 1.**
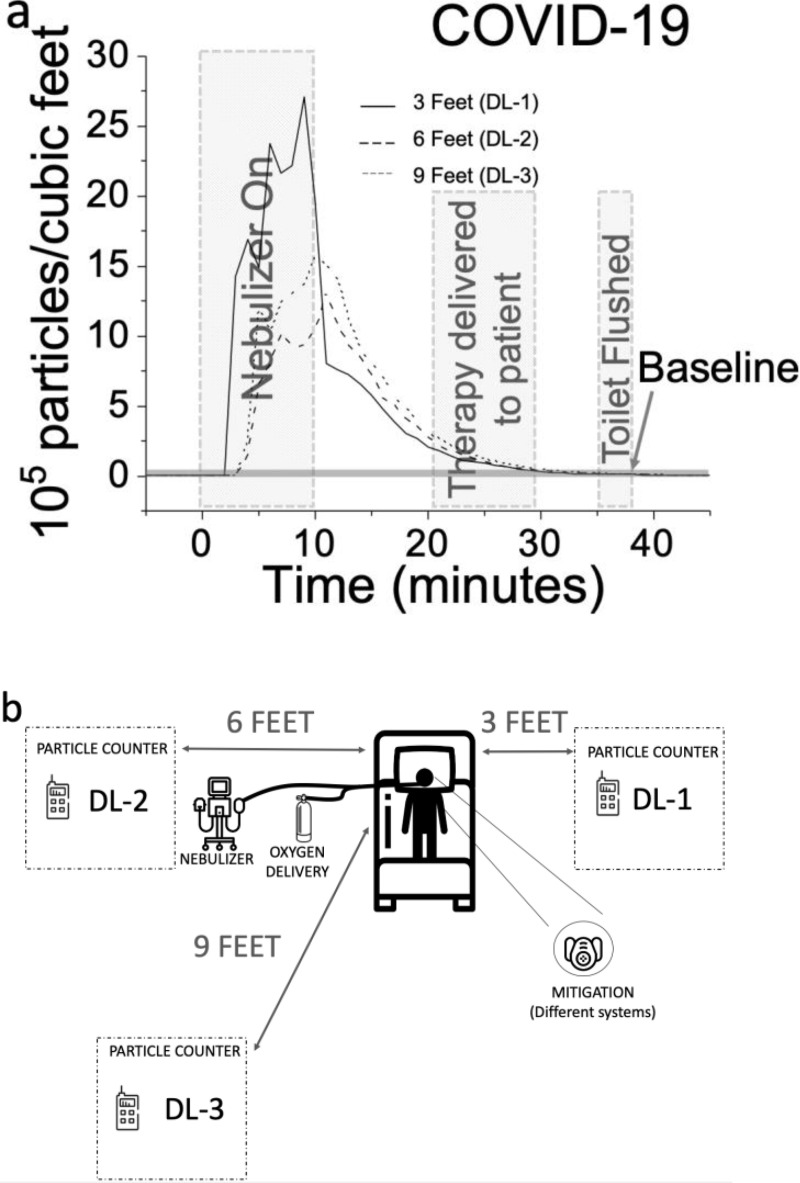
Nebulization Aerosol Dispersion. a) Particle count concentration during HFNC treatment with 21% of Oxygen, 30 L/min of air flow rate and nebulization with 3 ml of medicine in saline physiological solution to a COVID-19 patient, respiratory exercise therapy and toilet flushing activity. The aerosol particle count profiles are simultaneously taken at 3 different positions of: 3 feet (----), 6 feet (–––) and 9 feet (····) from the subject by an aerosol particle counter system (DL). The subject has no mitigation mask. b) Experimental design for testing the nebulization’s aerosol dispersion in the patient’s room (air exchange rate = 20 h^−1^).

Evaluation of all above-mentioned particle counters were performed in a sensing chamber using pure polystyrene particles (Bang Laboratories, Inc.) of size 0.19 μm suspended in ethanol. The particles were aerosolized with the mechanical nebulizer and introduce to a sensing chamber with the aid of a small fan integrated in the chamber inlet port. Different particle aerosol concentrations were created by aerosolizing different volumes from 0.1 mL to 3.0 mL. The sensitivity of the different particle counters to detect 0.19-μm polystyrene aerosol particles was evaluated and are shown in **Supplementary Information, Fig. 1A–B**. From all the particle counters evaluated, MET ONE HHPC2+ and Dylos DC1100 Pro demonstrated the capacity to detect 0.19 μm polystyrene particles with the highest sensitivity of all four counter, and therefore, they were the aerosol sensors of choice for the study.

For sake of simplification, we refer to MET ONE HHPC2+ particle counter as “MO sensor”, and to Dylos DC1100 and DC1700 as “DL sensor”.

### Experimental Rooms

Three different rooms were used in the study:

#### Simulation Room 1:

**Figure 2.**
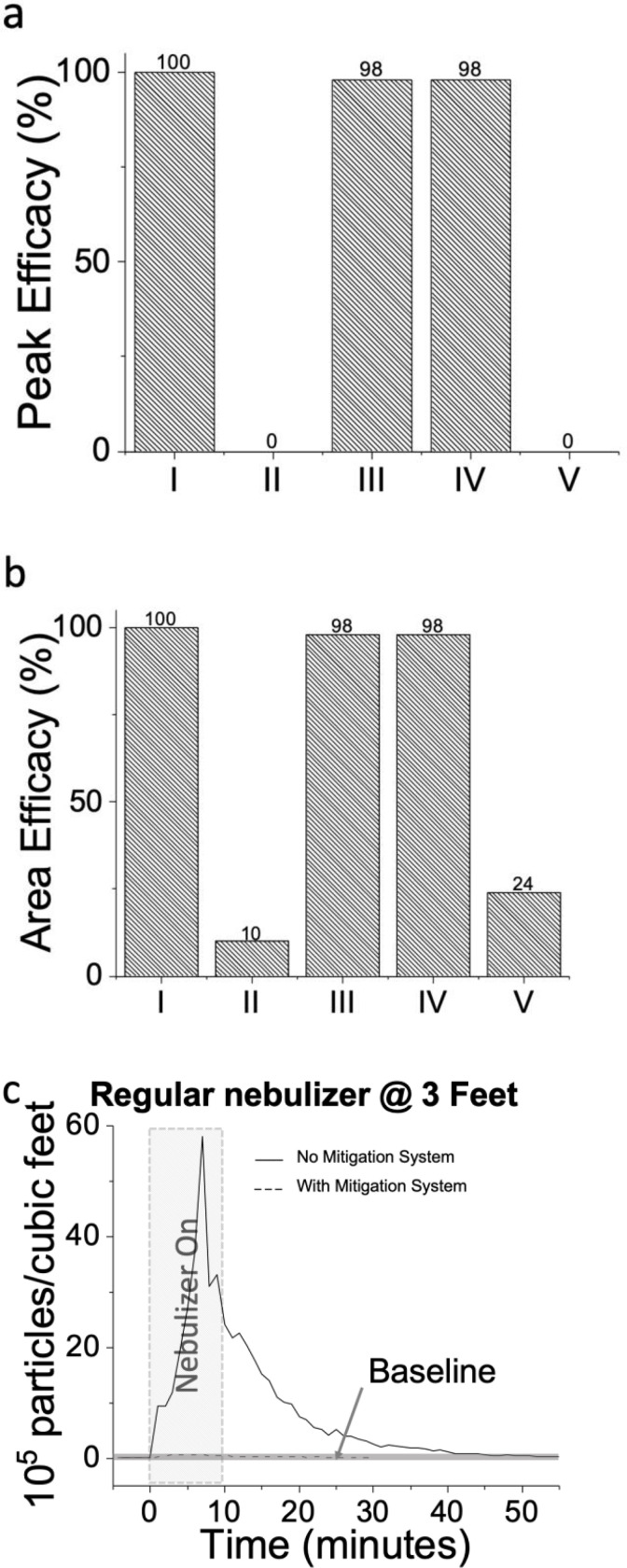
Efficacy of aerosol mitigation systems applied in this work during nebulization. Total volume of nebulization solution: 3 mL. The is efficacy for different study cases is assessed from particle count concentration vs. time profiles obtained during simulated nebulization therapies and includes **a)** peak analysis with (Eq. 1), **b)** area under the curve with (Eq. 2). Study cases correspond to (I): biofilter with fully sealed Full-Face Mask in BPAP oxygen therapy with filter, (II) surgical mask in HFNC, (III) modified silicone mask with biofilter and fan in HFNC, (IV) modified silicone mask with biofilter and fan in normal breathing, and (V) FFM with box mitigation. A piezoelectric nebulizer is used Cases I-III and V, and mechanical pump nebulizer is used in Case IV. c) Example of particle count concentration vs. time profiles assessed at distance of 3 feet from the subject during the simulated nebulization therapy for Case IV. Rectangular area at the bottom shows the room’s particle baseline levels. Aerosol level obtained in absence (----) and presence (----) of the mitigation system: modified silicone mask with biofilter and fan.

This experiment was carried in a room of 17’ × 13.6’ × 9’ designed to replicate an actual operation room equipped with a total of four or six sensors distributed as shown in **Supplementary Information, Fig. 2**: i) two sensors MO-1 and DL-1 at 3 feet distance, ii) two sensors MO-2 and DL-2 or one MO-2 at 6 feet and iii) two sensors MO-3 and DL-3 or DL-3 at 13 feet from the tested subject. The room had an air exchange rate of ~20 h^−1^. The measurements were taken every 1 minute. For MO sensors an integration time of 1 minute was used, for DL sensors an instant reading was registered every 1 minute. For each experiment a period of baseline was registered before starting the nebulization of 3 ml saline physiological solution, which was delivered through Piezoelectric Based Nebulizer: Pro-X Controller by Aerogen. Study cases I, II, III and V, which are typical healthcare facilities’ applications were tested in this room. The specifications of instruments used in these cases is indicated below.

#### Simulation Room 2:

This experiment was carried in a room 17’ × 13.6’ × 9’ with the same particle sensor distribution of simulation room 1. The room had an air exchange rate of ~2–3 h^−1^, which is typically representative of a home environment. Case IV including the nebulization of 3 ml saline physiological solution with a mechanical pump nebulizer: VH Complete Compressor by Veridian under normal breathing conditions was tested in this room. The measurements were taken every 1 minute. For MO sensors and integration time of 1 minute was used, for DL sensors an instant reading was registered every 1 minute. For each experiment a period of baseline was registered before starting the nebulization.

#### Patient Room:

This experiment was carried in a room of 17’ × 13.6’ × 9’ equipped with a small bathroom of 4’ × 6.25’ × 9’ in the back and an air ventilation rate, which is purposely set at 20 hour^−1^ to minimize transmission of the disease via aerosols. A total of three sensors was distributed as shown in Fig. 1b: one DL-1 sensor at 3 feet distance, one DL-2 sensor at 6 feet and one DL-3 sensor at 9 feet from the COVID-19 patient. The room had an air exchange rate of ~20 h^−1^. The 1-minute average reading were automatically recorded in the SD card of the instrument in the patient environment without disturbing the patient or putting the researchers at risk.

### Real and Simulated Human Subjects

A total of 6 human test subjects participated in this study. All methods discussed comply with the proper regulations for proper reporting experiments on human subjects through the Office for Human Research Protections with approved assurance of compliance via FederalWide Assurance (FWA number: ASU FWA 00009192). The human subjects were consented via ASU IRB: STUDY00006544 with the following IRB reviewers: Debra Murphy, empowered official and institutional official for IRB and Susan Metozky, IRB Compliance Office. In addition, we obtained informed consent from the subjects reported in the study for both study participation and publication in an online access publication. The human subjects were 22 to 55 years old, 5’5’’ to 6’ tall, and had body mass indexes < 25. They participated in of all of the tests in the study, except in tests corresponding to Case V, where a mannequin was used. The mannequin had an artificial lung simulator with a breath frequency of 16 breaths per m inute, Tidal Volume (Vt) of 400 to 1000 mL, and Ventilation (Ve) of 6 to 8 L/min.

### Validation test of the aerosol sensing instruments

**Figure 3.**
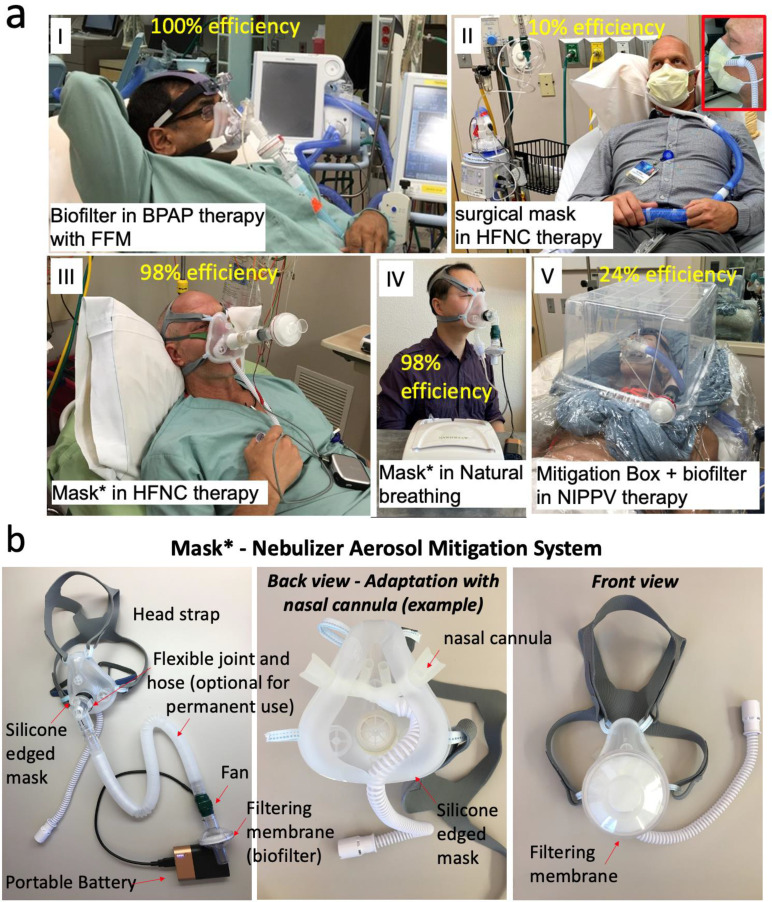
Different cases of combination of oxygen therapy and mitigation systems for nebulization. **a)** See text for description of Cases I-V. **b)** Silicone Mask* built for nebulization aerosol mitigation (see text for more details).

Before starting the testing with human subjects, and the different oxygen delivery and mitigation methods, three MO sensors and one DL sensor were positioned in the simulation room at 3, 6, and 13 feet from a piezoelectric nebulizer with 3 mL of 0.19 μm polystyrene particles solution in ethanol, and a nebulization was delivered to the mannequin with a non-invasive positive pressure ventilator (NIPPV) and no aerosol mitigation system connected to it. **Supplementary Information, Fig. 3A–B** show the corresponding particle concentration profiles, indicating a clear peak after the initiation of the nebulizer, which corroborated the capacity of MO and DL sensors to capture aerosol plumes from nebulization within a room (simulation room 1) with a high air exchange rate (20 h^−1^).

### Description of study cases

The application of the mitigation methods to different oxygen therapies rendered five study cases described below.

#### Case I:

The test subject used of a Full-Face Mask in bilevel positive airway pressure device (BPAP) therapy. The system was tested with and without the use of a biofilter located in its outlet (Fig. 3a–I). The nebulization therapy of 3 ml saline physiological solution was delivered through the Piezoelectric Based Nebulizer. The experiments were carried out in the simulation room 1 equipped with a total of four sensors: i) two sensors MO-1 and DL-1 at 3 feet distance, ii) one sensor MO-2 placed at 6 feet, and iii) one sensor MO-3 at 13 feet from the test subject. **Supplementary Information, Fig. 4A–C** show detailed particle concentration profiles over time assessed at baseline with no nebulization, with nebulization and no mitigation, and with nebulization and mitigation at different distances. **Supplementary Information, Fig. 4D** shows an overlapped particle concentration profiles over time assessed during nebulization and under no mitigation conditions, and a higher exposure to nebulizer aerosols are observed at 6 feet with respect to 3 feet location due to the air circulation streams in the room.

#### Case II:

The test subject used of a High Flow Nasal Cannula (HFNC) therapy, with and without a surgical mask as mitigation system (Fig. 3a–II). The nebulization therapy of 3 ml saline physiological solution was delivered through the Piezoelectric Based Nebulizer. The experiments were carried out in the simulation room 1 equipped with a total of four sensors: i) two sensors MO-1 and DL-1 at 3 feet distance, ii) one sensor MO-2 placed at 6 feet, and iii) one sensor MO-3 at 13 feet from the test subject. **Supplementary Information, Fig. 5A–C** show detailed particle concentration profiles over time assessed at baseline with no nebulization, with nebulization and no mitigation, and with nebulization and mitigation at different distances. **Supplementary Information, Fig. 5D–E** shows overlapped particle concentration profiles over time for all sensor locations without and with modified silicone mask + biofilter + fan use conditions, respectively. Similarly to case I, a higher exposure to nebulizer aerosols is observed at 6 feet with respect to 3 feet location for the mask mitigation condition due to the air circulation streams in the room.

#### Case III:

The test subject used of a High Flow Nasal Cannula (HFNC) therapy, with and without a modified silicone mask and a biofilter and fan as mitigation system (Fig. 3a–III). The nebulization therapy of 3 ml saline physiological solution was delivered through the Piezoelectric Based Nebulizer. The experiments were carried out in the simulation room 1 equipped with a total of six sensors: i) two sensors MO-1 and DL-1 at 3 feet distance, ii) one sensor MO-2 placed at 6 feet, and iii) one sensor MO-3 at 13 feet from the test subject. **Supplementary Information, Fig. 6A–C** show detailed particle concentration profiles over time assessed at baseline with no nebulization, with nebulization and no mitigation, and with nebulization and mitigation at different distances. **Supplementary Information, Fig. 6D–E** shows an overlapped particle concentration profiles over time for all sensor locations without and with modified silicone mask + biofilter + fan use conditions, respectively. **Supplementary Information, Fig. 6E** (case of use of the modified mask+ biofilter + fan) shows the clear reduction of the nebulizer aerosol particles close to the baseline levels.

#### Case IV:

The test subject was breathing naturally without the help of any oxygen therapy and was tested with and without the modified silicone mask + biofilter + fan as mitigation system (Fig. 3a–IV). The nebulization therapy of 3 ml saline physiological solution was delivered through the Mechanical Based Nebulizer. This nebulizer delivered a 4L/min air with the aerosol. The experiments were carried out in the simulation room 2 equipped with a total of six sensors: i) two sensors MO-1 and DL-1 at 3 feet distance, ii) two sensors MO-2 and DL-2 placed at 6 feet, and iii) two sensors MO-3 and DL-3 at 13 feet from the test subject. **Supplementary Information, Fig. 7A–C** show detailed particle concentration profiles over time assessed at baseline with no nebulization, with nebulization and no mitigation, and with nebulization and mitigation at different distances. **Supplementary Information, Fig. 7D–E** shows an overlapped particle concentration profiles over time for all sensor locations without and with modified silicone mask + biofilter + fan use conditions, respectively. **Supplementary Information, Fig. 7E** (case of use of the modified mask+ biofilter + fan.) shows the clear reduction of the nebulizer aerosol particles close to the baseline levels.

#### Case V:

The mannequin was ventilated with a Full-Face Mask connected to a Non-Invasive Positive Pressure Ventilator (NIPPV) and was tested with and without a mitigation box that had a biofilter attached to it as mitigation system (Fig. 3a–V). The mitigation box design was inspired on the isolation hood that has gained attention with the focus of mitigation SARS-CoV-2 pathogens^23^. The nebulization therapy of 3 ml saline physiological solution was delivered through the Piezoelectric Based Nebulizer. The experiments were carried out in the simulation room 1 equipped with a total of six sensors: i) two sensors MO-1 and DL-1 at 3 feet distance, ii) two sensors MO-2 and DL-2 placed at 6 feet, and iii) two sensors MO-3 and DL-3 at 13 feet from the test subject. **Supplementary Information, Fig. 8A–C** show detailed particle concentration profiles over time assessed at baseline with no nebulization, with nebulization and no mitigation, and with nebulization and mitigation at different distances. **Supplementary Information, Fig. 7D–E** shows an overlapped particle concentration profiles over time for all sensor locations with and without modified mitigation box, respectively.

### Oxygen Delivery Methods

The oxygen delivery methods practiced in this study included the following specifications:**BiPAP:** Full-Face Mask was used to deliver 21% of O_2_ and 30 L/min of air flow through BPAP ventilator to test subjects. Respironics V60 by Philips provided a non-invasive bilevel positive airway pressure breathing support. Amara Full-Face Mask by Philips was used.

#### HFNC:

Optiflow+ High flow nasal cannula (HFNC) from Fisher & Paykel was employed delivering 21% of O_2_ and 30 or 60 L/min was used.

### Aerosol Mitigation Methods

To mitigate the aerosol spread from the test subject the four different types of mitigations had the following specifications:

#### Biofilter:

ThermoFlo filter by Arc Medical or Hudson Bacterial/viral Filter were employed, as it provides 99.9% bacterial and viral efficiency.

#### Disposable Surgical Mask:

Common surgical mask used by doctors and patients.

#### Modified Silicone Disposable Mask:

A custom designed mask by altering the conventional mask by Breezing™ (Tempe. AZ). The mask is fitted with the high flow nasal cannula or the mechanical nebulizer outlet port by slicing the silicon paddings of Breezing™ mask. The mask is also integrated with a biofilter in the exhalation tube followed by a battery-operated fan (Mouser Electronics).

#### Box Mitigation:

A plastic box drilled two holes with each at opposite ends fitted with one-way air flow valve to provide one directional ventilation and this box is fitted with 2 MIL plastic curtains that runs throughout the test subject’s body to reduce the leak of aerosols.

### Assessment of air exchange rate of the rooms

The air flowing in and out of the room is an important parameter as it is a major factor affecting the dispersion of aerosol. Increasing air exchange rates improves the room’s capability of eliminating aerosols and can be determined by the declining slope of particles being vented out from the room^15^. To determine the air exchange rate in the tested room, the room was filled with CO_2_ gas, and the CO_2_ gas concentration was monitored over time with a carbon dioxide sensor (Telaire by GE). The declining slope of recorded CO_2_ levels yields air exchange rates of 20 hr^−1^ for the simulation room 1 and patient room, and 2–3 hr^−1^ for simulation room 2.

## Abbreviations

BPAP: Bilevel positive airway pressure
HFNC: High Flow Nasal Cannula
NIPPV: Non-invasive Positive Pressure Ventilator
FFM: Full-Face Mask
